# Supplementary material for: High-dimensional Biomarker Identification for Scalable and Interpretable Disease Prediction via Machine Learning Models
Source: bioRxiv. 2024 Oct 7:2024.10.04.616748. Preprint. [Version 1] doi: 10.1101/2024.10.04.616748 (PMC11482776; doi:10.1101/2024.10.04.616748)
Supplement: Supplement 1 [file media-1.pdf]

Supplementary Materials of "High-dimensional Biomarker Identification for Scalable and Interpretable Disease Prediction via Machine Learning Models"

Yifan Dai[1], Fei Zou[1,2], Baiming Zou[1,3*]

1 Department of Biostatistics, University of North Carolina at Chapel Hill

2 Department of Genetics, University of North Carolina at Chapel Hill

3 School of Nursing, University of North Carolina at Chapel Hill

* Corresponding author: bzou@email.unc.edu

Simulation Studies on Binary Outcomes

We conducted simulation studies on binary data generated from the following models, i.e., one for generalized linear impacts and the other for generalized nonlinear effects.

$$\begin{aligned}
\text{logit}[P(y|\mathbf{x})] &= \sum_{j=1}^{10} \beta_j x_j \\
\text{logit}[P(y|\mathbf{x})] &= \sum_{j=1}^{4} 4\sin(2x_j) - \sum_{j=5}^{8} 4\log(2x_j^2+1) + 2x_9\exp(x_{10}) + 13
\end{aligned}$$

where $\beta_j \sim \mathcal{U}(2,3)$. All other data structures are generated in the same way as in the continuous scenarios. Results are presented in Supplementary Figures 1 $\sim$ 4. Similar to the results for continuous outcomes, HFS effectively selects most causal features, while HiFIT further reduces noise features, thereby enhancing the prediction accuracy of machine learning models.

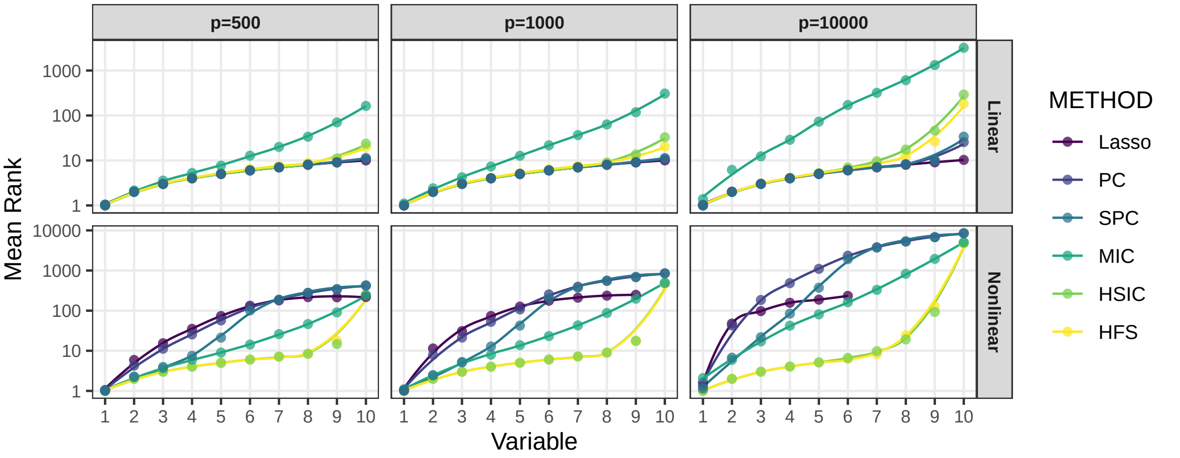

Supplementary Figure 1: **Average Rank of Causal Features Selected by Pre-Screening Methods on Binary Outcomes.**The x-axis denotes the number of selected causal features, and the corresponding value of the y-axis represents the average rank of this feature over 100 repetitions. The curves are generated by locally estimated scatterplot smoothing.

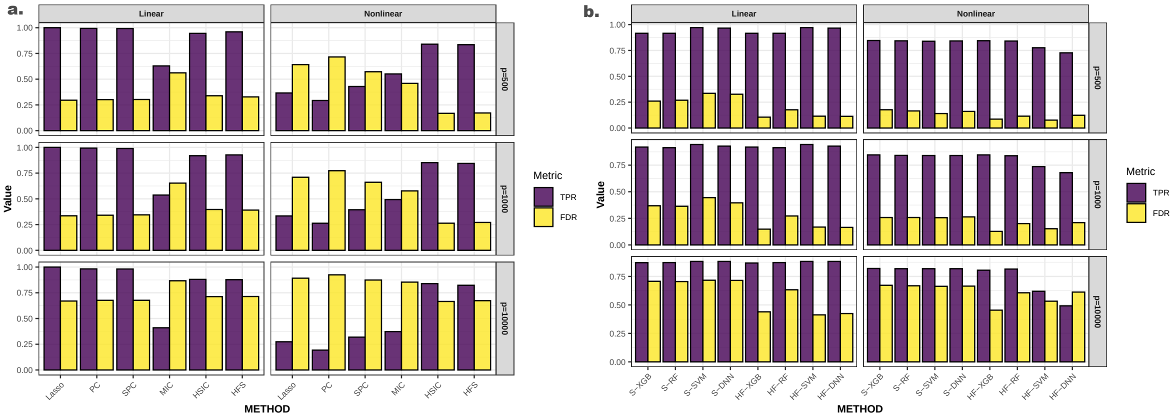

Supplementary Figure 2: **High-dimensional Feature Pre-Screening and Selection Results on Binary Outcomes.** **(a)** Performance of feature pre-screening methods. **(b)** Feature selection results of HiFIT models. TPR and FDR are averaged over 100 simulations.

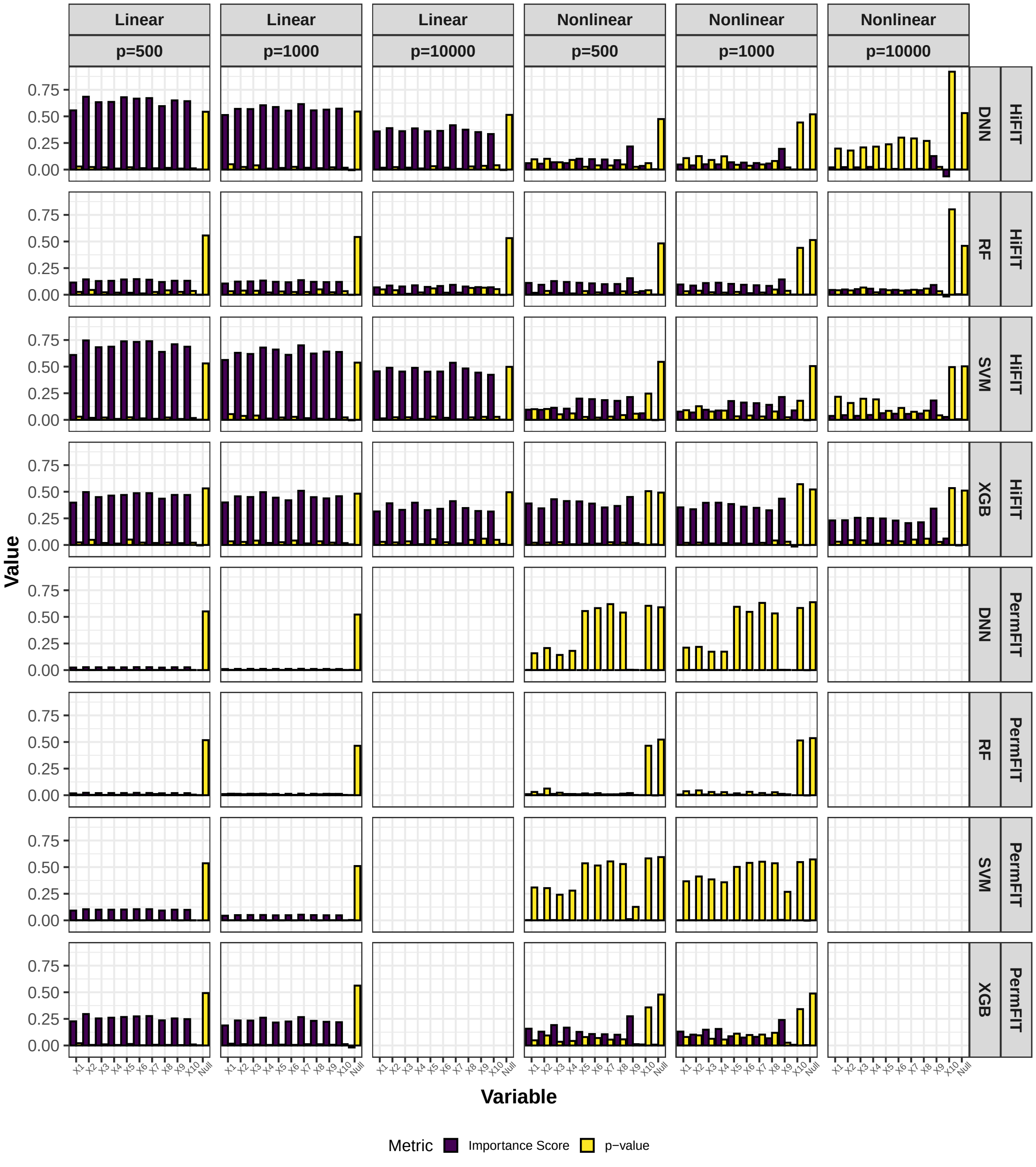

Supplementary Figure 3: **HiFIT Feature Interpretation Results on Binary Outcomes.** Average feature importance scores and p-values for 10 causal variables (denoted as $X_1, ..., X_{10}$) and the feature set of nuisance features (denoted as null) over 100 repetitions. Importance scores of features not selected by HFS are set to zero.

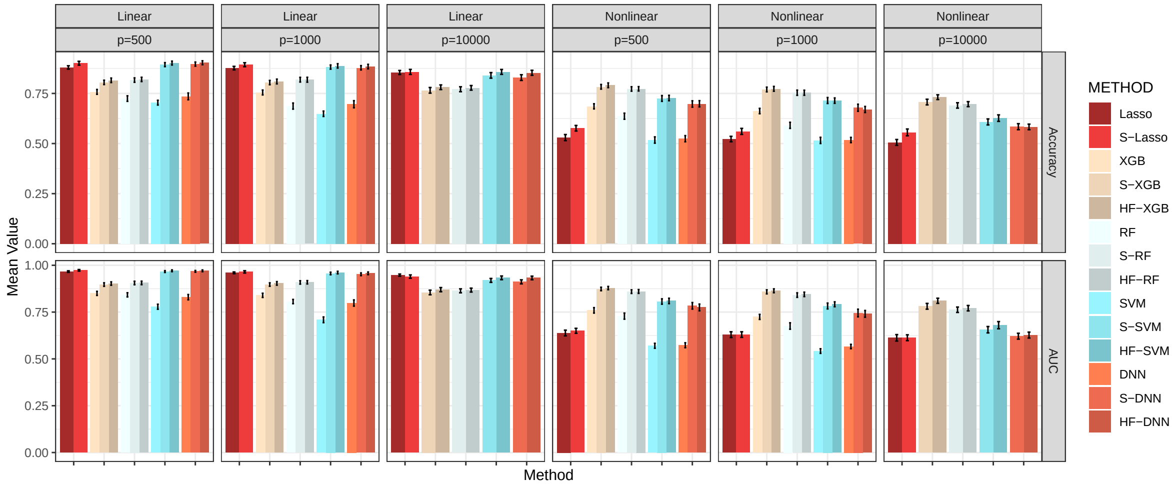

Supplementary Figure 4: **Average Accuracy and AUC for Methods in Comparison on Binary Outcomes.** Lasso, XGB, RF, SVM, and DNN: specific models with all features; S-Lasso, S-XGB, S-RF, S-SVM, S-DNN: specific models with HFS pre-screening; HF-XGB, HF-RF, HF-SVM, HF-DNN: specific models with HiFIT feature selection. Simulation in each scenario is repeated 100 times.
